# A survey of DNA motif finding algorithms

**DOI:** 10.1186/1471-2105-8-S7-S21

**Published:** 2007-11-01

**Authors:** Modan K Das, Ho-Kwok Dai

**Affiliations:** 1 Computer Science Department, Oklahoma State University, Stillwater, Oklahoma 74078, USA; 2 USDA-ARS, Department of Plant Sciences, University of Arizona, Tucson, Arizona 85721, USA

## Abstract

**Background:**

Unraveling the mechanisms that regulate gene expression is a major challenge in biology. An important task in this challenge is to identify regulatory elements, especially the binding sites in deoxyribonucleic acid (DNA) for transcription factors. These binding sites are short DNA segments that are called motifs. Recent advances in genome sequence availability and in high-throughput gene expression analysis technologies have allowed for the development of computational methods for motif finding. As a result, a large number of motif finding algorithms have been implemented and applied to various motif models over the past decade. This survey reviews the latest developments in DNA motif finding algorithms.

**Results:**

Earlier algorithms use promoter sequences of coregulated genes from single genome and search for statistically overrepresented motifs. Recent algorithms are designed to use phylogenetic footprinting or orthologous sequences and also an integrated approach where promoter sequences of coregulated genes and phylogenetic footprinting are used. All the algorithms studied have been reported to correctly detect the motifs that have been previously detected by laboratory experimental approaches, and some algorithms were able to find novel motifs. However, most of these motif finding algorithms have been shown to work successfully in yeast and other lower organisms, but perform significantly worse in higher organisms.

**Conclusion:**

Despite considerable efforts to date, DNA motif finding remains a complex challenge for biologists and computer scientists. Researchers have taken many different approaches in developing motif discovery tools and the progress made in this area of research is very encouraging. Performance comparison of different motif finding tools and identification of the best tools have proven to be a difficult task because tools are designed based on algorithms and motif models that are diverse and complex and our incomplete understanding of the biology of regulatory mechanism does not always provide adequate evaluation of underlying algorithms over motif models.

## Introduction

The gene is the fundamental unit of inherited information in deoxyribonucleic acid (DNA), and is defined as a section of base sequences that is used as a template for the copying process called transcription. The main idea in gene expression is that every gene contains the information to produce a protein. Gene expression begins with binding of multiple protein factors, known as transcription factors, to enhancer and promoter sequences. Transcription factors regulate the gene expression by activating or inhibiting the transcription machinery. Understanding the mechanisms that regulate gene expression is a major challenge in biology. Identifying regulatory elements, especially the binding sites in DNA for transcription factors is a major task in this challenge. Pattern discovery in DNA sequences is one of the most challenging problems in molecular biology and computer science. In its simplest form, the problem can be formulated as follows: given a set of sequences, find an unknown pattern that occurs frequently. If a pattern of *m *letters long appears exactly in every sequence, a simple enumeration of all *m*-letter patterns that appear in the sequences gives the solution. However, when one works with DNA sequences, it is not that simple because patterns include mutations, insertions or deletions of nucleotides.

A DNA motif is defined as a nucleic acid sequence pattern that has some biological significance such as being DNA binding sites for a regulatory protein, i.e., a transcription factor. Normally, the pattern is fairly short (5 to 20 base-pairs (bp) long) and is known to recur in different genes or several times within a gene [1]. DNA motifs are often associated with structural motifs found in proteins. Motifs can occur on both strands of DNA. Transcription factors indeed bind directly on the double-stranded DNA. Sequences could have zero, one, or multiple copies of a motif. In addition to the common forms of DNA motifs two special types of DNA motifs are recognized: palindromic motifs and spaced dyad (gapped) motifs. A palindromic motif is a subsequence that is exactly the same as its own reverse complement, e.g., CACGTG. A spaced dyad motif consists of two smaller conserved sites separated by a spacer (gap). The spacer occurs in the middle of the motif because the transcription factors bind as a dimer. This means that the transcription factor is made out of two subunits that have two separate contact points with the DNA sequence. The parts where the transcription factor binds to the DNA are conserved but are typically rather small (3–5 bp). These two contact points are separated by a non-conserved spacer. This spacer is mostly of fixed length but might be slightly variable.

Given a set of DNA sequences (promoter region), the motif finding problem is the task of detecting overrepresented motifs as well as conserved motifs from orthologous sequences that are good candidates for being transcription factor binding sites. A large number of algorithms for finding DNA motifs have been developed. Most of these algorithms are designed to deduce motifs by considering the regulatory region (promoter) of several coregulated genes from a single genome. It is assumed that coexpression of genes arises mainly from transcriptional coregulation. As coregulated genes are known to share some similarities in their regulatory mechanism, possibly at transcriptional level, their promoter regions might contain some common motifs that are binding sites for transcription factors. A sensible approach to detect these regulatory elements is to search for statistically overrepresented motifs in the promoter region of such a set of coexpressed genes. A statistically overrepresented motif means a motif that occurs more often than one would expect by chance. Therefore, these algorithms search for overrepresented motifs in this collection of promoter sequences. However, most of these motif finding algorithms have been shown to work successfully in yeast and other lower organisms, but perform significantly worse in higher organisms. To overcome this difficulty recent motif finding algorithms are taking advantage of cross-species genome comparison or phylogenetic footprinting [2]. The simple premise underlying phylogenetic footprinting is that selective pressure causes functional elements to evolve at a slower rate than non-functional sequences. This means that usually well conserved sites among a set of orthologous promoter regions are excellent candidates for functional regulatory elements or motifs. Several motif finding algorithms have been developed based on phylogenetic footprinting [3-8]. Most recently, algorithms that integrate DNA sequence data from coregulated genes and phylogenetic footprinting have significantly improved motif finding from genomic sequences [9-16]. Efforts have also focused toward developing algorithms that incorporate parameters that are useful for motif finding in higher organisms [17]. Stormo [18] presented an excellent history of development and application of computer algorithms for DNA motif finding. Since then a remarkably rapid development has occurred in DNA motif finding algorithms and a large number of DNA motif finding algorithms have been developed and published. In this survey, we review the recent developments in DNA motif finding algorithms.

## Motif discovery algorithms

Based on the type of DNA sequence information employed by the algorithm to deduce the motifs, we classify available motif finding algorithms into three major classes: (1) those that use promoter sequences from coregulated genes from a single genome, (2) those that use orthologous promoter sequences of a single gene from multiple species (i.e., phylogenetic footprinting) and (3) those that use promoter sequences of coregulated genes as well as phylogenetic footprinting. However, most of the earlier literature categorized motif finding algorithms into two major groups based on the combinatorial approach used in their design: (1) word-based (string-based) methods that mostly rely on exhaustive enumeration, i.e., counting and comparing oligonucleotide frequencies and (2) probabilistic sequence models where the model parameters are estimated using maximum-likelihood principle or Bayesian inference. The word-based enumerative methods guarantee global optimality and they are appropriate for short motifs and are therefore useful for motif finding in eukaryotic genomes where motifs are generally shorter than prokaryotes. The word-based methods can also be very fast when implemented with optimized data structures such as suffix trees [19] and are a good choice for finding totally constrained motifs, i.e., all instances are identical. However, for typical transcription factor motifs that often have several weakly constrained positions, word-based methods can be problematic and the result often needs to be post-processed with some clustering system [20]. Word-based methods also suffer from the problem of producing too many spurious motifs. The probabilistic approach involves representation of the motif model by a position weight matrix [21]. Position weight matrices are often visualized as a pictogram in which each position is represented by a stack of letters whose height is proportional to the information content of that position [22]. Probabilistic methods have the advantage of requiring few search parameters but rely on probabilistic models of the regulatory regions, which can be very sensitive with respect to small changes in the input data. Many of the algorithms developed from the probabilistic approach are designed to find longer or more general motifs than are required for transcription factor binding sites. Therefore, they are more appropriate for motif finding in prokaryotes, where the motifs are generally longer than eukaryotes. However, these algorithms are not guaranteed to find globally optimal solutions, since they employ some form of local search, such as Gibbs sampling, expectation maximization (EM) or greedy algorithms that may converge to a locally optimal solution.

A list of motif finding algorithms is presented chronologically in Additional file 1. Here we review some representative motif finding algorithms from the three major classes (based on the DNA sequence data employed) of algorithms described above.

## Algorithms based on promoter sequences of coregulated genes

Most of the earlier algorithms designed to find motifs use a set of promoter sequences of coregulated genes to identify statistically overrepresented motifs. The combinatorial approach (word-based, probabilistic, or other) underlying their design leads to a further classification.

### Word-based algorithms

van Helden *et al*. [23] developed the motif finding algorithm Oligo-Analysis based on the word-based approach. Although conceptually simple, their algorithm proved efficient for extracting motifs from most of the yeast (*Saccharomyces cerevisiae*) regulatory families they analyzed. These motifs had been previously found by laboratory experimental analysis. Furthermore, putative new regulatory sites were predicted within upstream regions of coregulated genes. In contrast with heuristic methods, this oligonucleotide analysis is rigorous and exhaustive. However, its range of detection is limited to relatively simple patterns that include short motifs with highly conserved core. The methodology used in developing the algorithm includes (1) constitution of regulatory families and (2) calculation of expected oligonucleotide frequencies. Later, van Helden *et al*. [24] extended their method to find spaced dyad motifs. Because the spacer can be different for distinct motifs, the spacer length is systematically varied between 0 and 16. The significance of this type of motif can be computed based on the combined score of the two conserved parts in the input data or based on the estimated complete dyad frequency from a background data set. The greatest shortcomings of the algorithm of van Helden *et al*. [23] is that there are no variations allowed within an oligonucleotide. Tompa [25] addressed this problem when he proposed an exact word-based method to find short motifs in DNA sequences. His algorithm was particularly applied to the ribosome binding site problem. Tompa took into account both the absolute number of occurrences and the background distribution and created a table that, for each *k*-mer (length-*k *sequence) *s*, records the number *N*_*s *_of sequences containing an occurrence of *s*, where an occurrence allows for a small, fixed number *c *of substitution residues in *s*. Then a reasonable measure of *s *as a motif would be based on how unlikely it is to have *N*_*s *_occurrences if the sequences were drawn at random according to the background distribution. The statistical significance test for motif occurrences used and described by Tompa [25] is as follows. Let *X *be a single random sequence of the specified length *L*, with residues drawn randomly and independently from the background distribution, or alternatively generated by a Markov chain according to the background dinucleotide distribution. Suppose that *p*_*s *_is the probability that *X *contains at least one occurrence of the *k*-mer *s*, allowing for *c *substitutions. Under the reasonable assumption that *N *length-*L *random sequences of *X *are independent, the expected number of containing at least one occurrence of *s *among the *N *random sequences is *Np*_*s*_, and its standard deviation is Nps(1−ps)
 MathType@MTEF@5@5@+=feaafiart1ev1aaatCvAUfKttLearuWrP9MDH5MBPbIqV92AaeXatLxBI9gBaebbnrfifHhDYfgasaacH8akY=wiFfYdH8Gipec8Eeeu0xXdbba9frFj0=OqFfea0dXdd9vqai=hGuQ8kuc9pgc9s8qqaq=dirpe0xb9q8qiLsFr0=vr0=vr0dc8meaabaqaciaacaGaaeqabaqabeGadaaakeaadaGcaaqaaiabd6eaojabdchaWnaaBaaaleaacqWGZbWCaeqaaOGaeiikaGIaeGymaeJaeyOeI0IaemiCaa3aaSbaaSqaaiabdohaZbqabaGccqGGPaqkaSqabaaaaa@3797@. Therefore, the associated *z-*score is

Ms=(Ns−Nps)/Nps(1−ps),
 MathType@MTEF@5@5@+=feaafiart1ev1aaatCvAUfKttLearuWrP9MDH5MBPbIqV92AaeXatLxBI9gBaebbnrfifHhDYfgasaacH8akY=wiFfYdH8Gipec8Eeeu0xXdbba9frFj0=OqFfea0dXdd9vqai=hGuQ8kuc9pgc9s8qqaq=dirpe0xb9q8qiLsFr0=vr0=vr0dc8meaabaqaciaacaGaaeqabaqabeGadaaakeaacqWGnbqtdaWgaaWcbaGaem4Camhabeaakiabg2da9iabcIcaOiabd6eaonaaBaaaleaacqWGZbWCaeqaaOGaeyOeI0IaemOta4KaemiCaa3aaSbaaSqaaiabdohaZbqabaGccqGGPaqkcqGGVaWldaGcaaqaaiabd6eaojabdchaWnaaBaaaleaacqWGZbWCaeqaaOGaeiikaGIaeGymaeJaeyOeI0IaemiCaa3aaSbaaSqaaiabdohaZbqabaGccqGGPaqkaSqabaGccqGGSaalaaa@46D1@

where *M*_*s *_is the number of standard deviations by which the observed value *N*_*s *_exceeds its expectation, and is sometimes called the "*z-*score", "normal deviate", or deviation in standard units. The random quantity of *M*_*s *_is asymptotically normally distributed, and normalized to have mean 0 and standard deviation 1, making it suitable for comparing different motifs *s*. Tompa proposed an efficient algorithm to estimate *p*_*s *_from a set of background sequences based on a Markov chain.

Using a similar approach, Sinha and Tompa [26] developed the algorithm YMF (Yeast Motif Finder). They derived the motif model from a study of known transcription factor binding sites in yeast. The inputs to the algorithm are a set of upstream sequences, the number of non-spacer characters in the motifs to be enumerated, and the transition matrix for an order *m *Markov chain constructed from the full complement of upstream sequences of yeast. This algorithm is guaranteed to produce motifs with greatest z-score (described above). They conducted a validation experiment where YMF was used to identify candidate binding sites in 23 well studied regulons (a set of genes controlled by a common regulator) of yeast. For 18 of these regulons, YMF succeeded in reporting the known binding site consensus for the regulon's principal transcription factor. They also applied YMF for motif finding from gene families in the functional and mutant phenotype catalogues of *S. cerevisiae *from MIPS database [27] and found many promising novel transcription factor binding sites. Sinha and Tompa [28] performed experiments using synthetic and yeast promoter sequences to compare the performance of YMF to the algorithms MEME [29] and AlignACE [30]. They reported that YMF made more accurate predictions of known regulatory elements on more of the yeast regulons than the other two tools. However, from this study they also concluded that it may be beneficial to try a few very different motif discovery tools in addition to YMF because of their observance that different tools performed better with different data sets.

Brazma *et al*. [31] used a word-based approach to develop a motif finding algorithm that looks for occurrences of regular expression-type patterns. They applied the algorithm for discovering (1) patterns in the complete set of over 6000 sequences taken from upstream of the putative yeast genes and (2) patterns in the upstream regions of coregulated genes in yeast. Among the highest rating patterns, most had matches to known motifs in yeast.

Sagot [19] introduced a word-based approach for motif finding that is based on the representation of a set of sequences with a suffix tree. Vanet *et al*. [32] used suffix trees to search for single motifs in whole genomes of bacteria. Marsan and Sagot [33] extended this method to search for combinations of motifs. Representation of upstream sequences as suffix trees gave a large number of possible combination, however, the implementation was still efficient. The motif finding algorithms, Weeder and MITRA (Mismatch Tree Algorithm), developed by Pavesi *et al*. [34] and Eskin and Pevzner [35] respectively, are also based on the suffix tree and its variant. The algorithms WINNOWER [36] and cWINNOWER [37] uses word-based approach combined with graph-theoretic methods for motif finding.

### Probabilistic algorithms

One of the first implementations for finding a matrix representation of transcription factor binding sites was a greedy probabilistic sequence model-based algorithm by Hertz *et al*. [38] to find the site with the highest information content. They used this algorithm to identify a common motif that was present once in every sequence. This algorithm has been substantially improved over the years and in their latest implementation of this algorithm (Consensus) Hertz and Stormo [39] provided a method to estimate the statistical significance of a given information content score based on large deviation statistics.

Down and Hubbard [40] developed the motif finding algorithm NestedMICA based on a probabilistic approach. This algorithm uses a sequence model based on an independent component analysis framework to learn models for multiple motifs simultaneously and it also uses an alternative inference strategy that is likely to find a globally optimal model in a single run. The authors tested the performance of this algorithm with the algorithm MEME on synthetic data and a well-characterized set of muscle regulatory regions and reported that NetsedMICA was more sensitive than MEME and in one case it successfully extracted a target motif from background sequence four times longer than could be handled by MEME.

Most probabilistic motif finding algorithms apply potent statistical techniques such as EM and Gibbs sampling and its extensions.

#### EM methods

EM for motif finding was introduced by Lawrence and Reilly [41] and it was an extension of the greedy algorithm for motif finding by Hertz *et al*. [38]. It was primarily developed for protein motifs, however, it can also be applied for DNA motif finding. No alignment of the sites is required and the basic model assumption is that each sequence must contain at least one common site. The uncertainty in the location of the sites is handled by employing the missing information principle to develop an EM algorithm. This approach allows for the simultaneous identification of the sites and characterization of the binding motifs. The MEME algorithm by Bailey and Elkan [29] extended the EM algorithm for identifying motifs in unaligned biopolymer sequences. The aim of MEME is to discover new motifs in a set of biopolymer sequences where little is known in advance about any motifs that may be present. MEME incorporated three novel ideas for discovering motifs. First, subsequences that actually occur in the biopolymer sequences are used as starting points for the EM algorithm to increase the probability of finding globally optimum motifs. Second, the assumption that each sequence contains exactly one occurrence of the shared motif is removed. Third, a method for probabilistically erasing shared motifs after they are found is incorporated so that several distinct motifs can be found in the same set of sequences, both when different motifs appear in different sequences and when a single sequence may contain multiple motifs.

#### Gibbs sampling methods

Among the probabilistic methods Gibbs sampling method has been used extensively for motif finding algorithms. Here we present a short description of the original Gibbs sampler for motif finding developed by Lawrence *et al*. [42]. They did not apply this algorithm to DNA sequence but applied to protein sequence in the original article. Since one of the original assumptions of this algorithm was that there exists at least one instance of a motif in every sequence, the method is sometimes called the "site sampler". Gibbs sampler is a Markov Chain Monte Carlo (MCMC) approach: "Markov-Chain", since the results from every step depends only on the results of the preceding one like in EM; "Monte-Carlo", since the way to select the next step is not deterministic but rather based on random sampling, i.e., random-numbers. The statistical background of MCMC methods is explained in the book by Liu [43] and that of Gibbs sampling in the article by Liu *et al*. [44]. In this algorithm it is assumed that we are given a set of *N *sequences *S*_1_,...,*S*_*N *_and we seek within each sequence mutually similar segments of specified width *W*. The algorithm maintains two evolving data structures. The first is the pattern description, in the form of a probabilistic model of residue frequencies for each position *i *from 1 to *W*, and consisting of the variables *q*_*i*,1_,...,*q*_*i*,20_, indexed by *W *positions and the 20 possible residues. This pattern description is accompanied by an analogous probabilistic description of the "background frequencies" *p*_1_,...,*p*_20 _with which residues occur in sites not described by the pattern. The second data structure, constituting the alignment, is a set of positions *a*_*k*_, for *k *from 1 to *N*, for the common pattern within the sequences. The objective will be to identify the "best", defined as the most probable, common pattern. This pattern is obtained by locating the alignment that maximizes the ratio of the corresponding pattern probability to background probability.

The algorithm is initialized by choosing random starting positions within the various sequences. It then proceeds through many iterations to execute the following two steps of the Gibbs sampler. (1) Predictive update step: One of the *N *sequences, *z*, is chosen either at random or in specified order. The pattern description *q*_*i*,*j *_and background frequencies *p*_*j *_are then calculated, as described below, from the current positions *a*_*k *_in all sequences excluding *z*. (2) Sampling step: Every possible segment of width *W *within sequence *z *is considered as a possible instance of the pattern. The probabilities *Q*_*x *_of generating each segment *x *according to the current pattern probabilities *q*_*i*,*j *_are calculated, as are the probabilities *P*_*x *_of generating these segments by the background probabilities *p*_*j*_. The weight *A*_*x *_= *Q*_*x*_/*P*_*x *_is assigned to segment *x*, and with each segment so weighted, a random one is selected (segment *x *is chosen with probability *A*_*x*_/∑_*j*_*A*_*j*_, where the sum is taken over all possible segments). Its position then becomes the new *a*_*z*_. This simple iterative procedure constitutes the basic algorithm.

The central idea is that the more accurate the pattern description constructed in step 1, the more accurate determination of its location in step 2, and vice versa. Given random position *a*_*k*_, in step 2 the pattern description *q*_*i*,*j *_will tend to favor no particular segment. Once some correct *a*_*k *_have been selected by chance, however, the *q*_*i*,*j *_begin to reflect, even though imperfectly, a pattern extant within other sequences. This process tends to recruit further correct *a*_*k*_, which in turn improves the discriminating power of the evolving pattern. An aspect of the algorithm alluded to in step 1 above concerns the calculation of the *q*_*i*,*j *_from the current set of *a*_*k*_. For the *i*th position of the pattern we have *N *- 1 observed amino acids, because sequence *z *has been excluded; let *c*_*i*,*j *_be the count of amino acid *j *in this position. Bayesian statistical analysis suggests that, for the purpose of pattern estimation, these *c*_*i*,*j*_'s should be supplemented with residue-dependent "pseudocounts" *b*_*j *_to yield pattern probabilities *q*_*i*,*j *_= (*c*_*i*,*j *_+ *b*_*j*_)/(*N *- 1 + *B*), where *B *is the sum of the *b*_*j*_. The *p*_*j *_are calculated analogously, with the corresponding counts taken over all non-pattern positions. After normalization, *A*_*x *_gives the probability that the pattern in sequence *z *belongs at position *x*. The algorithm finds the most probable alignment by selecting a set of *a*_*k*_'s that maximizes the product of this ratio. Equivalently, one may maximize *F*, the sum of the logarithms of these ratios. In the notation developed above, *F *is given by the formula

F=∑i=1W∑j=120ci,jlog⁡qi,jpj
 MathType@MTEF@5@5@+=feaafiart1ev1aaatCvAUfKttLearuWrP9MDH5MBPbIqV92AaeXatLxBI9gBaebbnrfifHhDYfgasaacH8akY=wiFfYdH8Gipec8Eeeu0xXdbba9frFj0=OqFfea0dXdd9vqai=hGuQ8kuc9pgc9s8qqaq=dirpe0xb9q8qiLsFr0=vr0=vr0dc8meaabaqaciaacaGaaeqabaqabeGadaaakeaacqWGgbGrcqGH9aqpdaaeWbqaamaaqahabaGaem4yam2aaSbaaSqaaiabdMgaPjabcYcaSiabdQgaQbqabaGccyGGSbaBcqGGVbWBcqGGNbWzdaWcbaWcbaGaemyCae3aaSbaaWqaaiabdMgaPjabcYcaSiabdQgaQbqabaaaleaacqWGWbaCdaWgaaadbaGaemOAaOgabeaaaaaaleaacqWGQbGAcqGH9aqpcqaIXaqmaeaacqaIYaGmcqaIWaama0GaeyyeIuoaaSqaaiabdMgaPjabg2da9iabigdaXaqaaiabdEfaxbqdcqGHris5aaaa@4E8B@

where the *c*_*i*,*j *_and *q*_*i*,*j *_are calculated from the complete alignment of the sequences.

#### Extensions to Gibbs sampling method

Based on Gibbs sampling strategy, Roth *et al*. [30] developed the motif finding algorithm AlignACE (Aligns Nucleic Acid Conserved Elements). This algorithm returns a series of motifs as weight matrices that are overrepresented in the input set of DNA sequences. In this algorithm, a motif is defined as the characteristic base-frequency patterns of the most information-rich columns of a set of aligned sites. It differs from the original Gibbs sampling algorithm [42] in the following major features. (1) The motif model is changed so that the base frequencies for non-site sequence are fixed according to the source genome (e.g., 62% A+T in the case of yeast). (2) Both strands of the input sequence are simultaneously considered at each step of the algorithm and overlapping sites are not allowed even if the sites are on opposite strands. (3) Simultaneous multiple motif searching is replaced by an approach in which single motifs are found and iteratively masked. (4) It uses an improved near-optimum sampling method. AlignACE uses the MAP (maximum *a priori *log-likelihood) score to judge different motifs sampled. It is a measure of the degree of overrepresentation of a motif as compared to the expected random occurrence of that motif in the sequence under consideration. The main drawback of MAP score used by this algorithm is the fact that some motifs occurring ubiquitously in a genome (e.g., A-rich motifs in yeast) are scored very highly, but are not likely to be relevant to the specific set of genes in question. Later, Hughes *et al*. [45] used AlignACE for finding motifs from groups of functionally related genes in yeast. Rather than applying the MAP for scoring motifs, they used an improved measure known as group specificity. This new measure takes into account the sequence of the entire genome and highlights those motifs that are found preferentially in association with the genes under consideration. Using this algorithm with the improved scoring technique they were able to find motifs that were previously identified as well as novel motifs.

Thijs *et al*. [46] developed the motif finding algorithm MotifSampler using a modification of the original Gibbs sampling algorithm. The two major modifications are (1) the use of a probability distribution to estimate the number of copies of the motif in a sequence and (2) the incorporation of a higher-order Markov-chain background model. The authors tested their algorithm on several data sets. For the data sets involving sequences from plants containing the G-box motif and the upstream sequences from bacterial genes regulated by oxygen-responsive protein FNR, MotifSampler was able to find expected motifs. From the experiment involving four clusters of coexpressed genes, expressed in response to wounding in *Arabidopsis thaliana*, they found several putative motifs that are related to the pathways involved in the plant defense mechanism.

Using a Gibbs sampling strategy, Liu *et al*. [47] developed the motif finding algorithm BioProspector that uses the promoter regions of coregulated genes. It differs from the original Gibbs sampler in the following points. (1) It uses zero to third-order Markov background models whose parameters are either given by the users or estimated from a specified sequence file. (2) The significance of each motif is judged based on a motif score distribution estimated by a Monte Carlo method. (3) It allows for the modeling of spaced dyad motifs and motifs with palindromic patterns. The authors of this algorithm were successful in finding motifs for binding of RAP1 protein in yeast, TATA-box motif in *Bacillus subtilis *and CRP protein binding site in *Escherichia coli*.

Shida [48] developed the motif discovery algorithm GibbsST using the method of simulated tempering with Gibbs sampling. Gibbs sampling is one of the most promising pattern discovery methods in terms of its flexibility and wide range of application, however, it is known to be rather strongly affected by the local optima problem [49]. Therefore, the Gibbs sampling method can be further improved by a search method in the solution space. In pattern discovery and bioinformatics in general, the simulated annealing method is mostly used for improvement of search methods in the solution space [50-52]. However, satisfactory improvements were not obtained using this method [48]. Simulated tempering is one of many proposals from the field of thermodynamics for the systematic avoidance of local optima in multivariate optimization problems and is quite useful for reducing the vulnerability of Gibbs sampling to local optima [48]. In the GibbsST algorithm the authors have utilized the simulated tempering approach to improve the Gibbs sampling method of motif finding. They validated this algorithm using synthetic data and actual promoter sequences extracted from yeast and obtained much increased resistance to the local optima problem.

### Machine learning techniques

Liu *et al*. [53] developed the algorithm FMGA based on genetic algorithms (GAs) for finding potential motifs in the regions located from the -2000 bp upstream to +1000 bp downstream of the transcription start site. The mutation in GA is performed by using position weight matrices to reserve the completely conserved positions. The crossover is implemented with specially designed gap penalties to produce the optimal child pattern. This algorithm also uses a rearrangement method based on position weight matrices to avoid the presence of a very stable local minimum, which may make it quite difficult for the other operators to generate the optimal pattern. The authors reported that FMGA performs better in comparison to MEME and Gibbs sampler algorithms.

Liu *et al*. [54] developed a self-organizing neural network structure for motif finding in DNA and protein sequences. The network contains several layers with each layer performing classifications at different level. The authors maintained a low computational complexity through the use of layered structure so that each pattern's classification is performed with respect to a small subspace of the whole input space. The authors also maintain a high reliability of their search algorithm using self-organizing neural network since it will grow as needed to make sure that all input patterns are considered and are given the same amount of attention. From simulation results the authors reported that their algorithm outperformed the algorithms MEME and Gibbs Sampler in certain aspects and their algorithm also works well for long DNA sequences.

### Other approaches

Kingsford *et al*. [55] used a mathematical programming approach for DNA motif discovery that involved finding subsequences of a given length such that the sum of their pairwise distances was minimized. They used integer linear programming (ILP) that utilizes the discrete nature of the distance metric imposed on pairs of subsequences. Since finding a solution to the ILP is computationally difficult, the authors tightened the linear programming relaxation by adding an exponential set of constraints and used an efficient separation algorithm that can find violated constraints and thus having a polynomial time solution. The authors tested the effectiveness of their approach in identifying DNA motifs in *E. coli *and demonstrated that the performance of their method is competitive with some Gibbs sampling-based algorithms for motif finding.

Kaplan *et al*. [56] used a structure-based approach for motif finding with no prior DNA binding data. They combine DNA sequence data and transcription factor protein structural information to infer context specific amino acid-nucleotide recognition preferences. This information is used to predict binding sites for novel transcription factors from the same structural family. The authors used Cys_2_His_2 _Zinc Finger protein family and showed that the learned DNA-recognition preferences are compatible with experimental results.

While many motif finding algorithms have been shown to work successfully in yeast and other organisms, most perform significantly worse in higher organisms [57]. Hon and Jain [17] developed a deterministic motif finding algorithm with application to the human genome. This deterministic method depends on an indexing technique to optimize the search process. The fast search procedure is coupled to a very simple scoring function that combines a preference for conservation among input sequences with a preference for under-represented sequences relative to the genome.

### Ensemble algorithm

Hu *et al*. [58] introduced the ensemble approach for motif finding to improve the prediction accuracy of the motif finding algorithms. They developed a novel clustering-based ensemble algorithm named EMD [59] for motif discovery by combining multiple predictions from multiple runs of one or more base component algorithms. The potential of an EMD algorithm lies in the fact that it could take advantage of superb predictions of every component algorithm. The authors used five component algorithms namely AlignACE, Bioprospector, MDScan [60], MEME and MotifSampler in their study. They tested their algorithm on a benchmark dataset generated from *E. coli *RegulonDB. The EMD algorithm achieved 22.4% improvement in terms of the nucleotide level prediction accuracy over the best stand-alone component algorithm. The authors pointed out that the advantage of the EMD algorithm is more significant for shorter input sequences, but most importantly, it always outperformed or at least stayed at the same performance level of the stand-alone component algorithms even for longer sequences.

## Algorithms based on phylogenetic footprinting

The major advantage of phylogenetic footprinting over the coregulated genes approach is that the latter requires a reliable method for identifying coregulated genes. Where as, using phylogenetic footprting approach, it is possible to identify motifs specific to even a single gene, as long as they are sufficiently conserved across the many orthologous sequences considered. The rapid accumulation of genomic sequences from a wide variety of organisms makes it possible to use the phylogenetic footprinting approach for motif finding. The standard method used for phylogenetic footprinting is to construct a global multiple alignment of the orthologous promoter sequences and then identify conserved region in the alignment using a tool such as CLUSTAL W [61]. However, it has been observed [3,4,62] that this approach to phylogenetic footprinting does not always work. The reason is that if the species are too closely related, the sequence alignment is obvious but uninformative, since the functional elements are not sufficiently better conserved than the surrounding nonfunctional sequence. On the other hand, if the species are too distantly related, it is difficult or impossible to find an accurate alignment. To overcome this problem, one of the several existing motif finding algorithms such as MEME, Consensus, Gibbs sampler have been used for phylogenetic footprinting. Cliften *et al*. [3] used AlignACE for motif finding by comparative DNA sequence analysis of several species of *Saccharomyces *and reported some successes where the global multiple alignment tools failed. McCue *et al*. [63] used Gibbs sampler for motif finding using phylogenetic footprinting in proteobacterial genomes. That the use of such general motif discovery algorithms can be problematic in phylogenetic footprinting has been pointed out by Blanchette and Tompa [4]. These motif finding algorithms do not take into account the phylogenetic relationship of the given sequences since these methods assume the input sequences to be independent. Therefore, the data sets containing a mixture of some closely related species will have an unduly high weight in the choice of motifs reported. Even if these methods were modified to weigh the input sequences unequally, this would still not capture the information in an arbitrary phylogenetic tree. To overcome this problem, Blanchette and Tompa [4] designed an algorithm for motif finding from phylogenetic footprinting that uses dynamic programming to find most parsimonious *k*-mer from each of the input sequences where *k *is the motif length.

Berezikov *et al*. [5] reported the motif finding algorithm CONREAL based on phylogenetic footprinting. This algorithm uses potential motifs as represented by positional weight matrices to establish anchors between orthologous sequences and to guide promoter sequence alignment. They compared the performance of CONREAL with two global alignment programs LAGAN [64] and AVID [65] using a reference data set and observed that CONREAL worked equally well for closely related species like rodent and human and has a clear added value for aligning promoter elements of more divergent species like human and fish, as it identifies conserved transcription factor binding sites that are not found by other methods.

Cliften *et al*. [6] used the phylogenetic footprinting approach to find motifs in *Saccharomyces *genomes. They searched for phylogenetic footprints among the genome sequences of six *Saccharomyces *species using the sequence alignment tool CLUSTAL W. Using this simple alignment technique they were able to find many statistically significant conserved sequence motifs. This was possible because they compared multiple genome sequences that are as optimally diverged as possible.

Wang and Stormo [7] developed the algorithm PHYLONET that systematically identifies phylogenetically conserved motifs by analyzing all of the promoter sequences of several related genomes and defines a network of regulatory sites for the organism. This algorithm involves construction of phylogenetic profiles for each promoter and then uses a BLAST-like algorithm to efficiently search through the entire profile space of all the promoters in the genome to identify conserved motifs and the promoters that contain them. Statistical significance of motifs is estimated by modified Karlin-Altschul statistics [66]. The authors used this algorithm to the analysis of 3524 yeast promoters and identified a highly organized regulatory network involving 3315 promoters and 296 motifs. This network includes nearly all of the currently known motifs and cover more than 90% of known transcription factor binding sites. They claimed that this algorithm can be applied to much larger genome such as the human genome.

Carmack *et al*. [8] developed a scanning algorithm, PhyloScan, which combines evidence from matching sites found in orthologous data from several related species with evidence from multiple sites within an intergenic region, to better detect regulons. The orthologous sequence data may be multiply aligned, unaligned, or a combination of aligned and unaligned. In aligned data, PhyloScan statistically accounts for the phylogenetic dependence of the species contributing data to the alignment and, in unaligned data, the evidence for sites is combined assuming phylogenetic independence of the species. The authors applied this algorithm to real sequence data from seven *Enterobacteriales *species and identified novel transcription factor binding motifs.

## Algorithms based on promoter sequences of coregulated genes and phylogenetic footprinting

These algorithms integrate two important aspects of a motif's significance, i.e., overrepresentation and cross-species conservation, into one probabilistic score. Gelfand *et al*. [9] used promoters of coregulated genes and orthologous promoter sequence data for finding overrepresented motifs in Archaea. They used the Smith-Waterman algorithm for signal identification, construction of recognition profiles, identification of candidate signals in genome sequences and protein similarity searches. In this study, they treated all data, i.e., coregulated and orthologous sequences as independent in spite the fact that the orthologous sequences are directly related. A similar treatment of the mixed set of data was done by the algorithm of McGuire *et al*. [10]. An algorithm by Kellis *et al*. [11] extracts motifs in two steps from the mixed data of two types of sequences. In the first step this algorithm finds a set of highly conserved motifs and in the second step the overrepresented motifs are extracted from this set. Prakash *et al*. [12] developed the algorithm OrthoMEME based on an EM approach that uses mixed data of two types of sequences. This algorithm searches the space of motifs and motif alignments simultaneously. Each motif occurrence is assumed to have an orthologous copy in the other species, which could be located anywhere in the corresponding promoter. The algorithm OrthoMEME is designed to handle orthologous sequences from two species.

Based on the Consensus algorithm [38] Wang and Stormo [13] developed the motif finding algorithm PhyloCon (Phylogenetic Consensus) that takes into account both conservation among orthologous genes and coregulation of genes within a species. This algorithm first aligns conserved regions of orthologous sequences into multiple sequence alignments or profiles, and then compares profiles representing non-orthologous sequences. Motifs emerge as common regions in these profiles. They presented a novel statistic to compare profiles of DNA sequences and a greedy approach to search for common subprofiles. They demonstrated that PhyloCon performed well on both synthetic and biological data. The strengths of PhyloCon are that it does not consider a single instance of a motif as a string of letters. Instead, it sees any position of such an instance as a probabilistic distribution over all possible nucleotides. Random mutations that could disrupt the significance of any copy of the motif are much less devastating to a probabilistic profile. Spurious random profiles are much less likely than spurious random motifs. Thus PhyloCon has a low false positive rate and is very tolerant of background sequence length. Extended background genomic conservation beyond the motif helps not only to reduce search space, but also to correctly align conserved motifs. By saving suboptimal alignments and comparing all of them, PhyloCon reduces its false negative rate.

Sinha *et al*. [14] developed the algorithm PhyME based on a probabilistic approach that handles data from promoters of coregulated genes and orthologous sequences. An important feature of this algorithm is that it allows motifs to occur in conserved as well as non-conserved regions in orthologous promoters, treating the two kinds of occurrences differently when scoring a motif. It does not require each binding site occurrence in one promoter to have an orthologous occurrence in any or all other species. This allows some flexibility in terms of the evolutionary distances spanned by the input sequences. For example, using a distantly related ortholog will help pinpoint motifs located in conserved regions but will not hamper the discovery of motifs absent from that ortholog. The authors evaluated this algorithm on synthetic data and data sets from yeast, fly and human. They compared PhyME with the algorithms MEME, OrthoMEME, PhyloGibbs [16], EMnEm [15] and GIBBS (Wadsworth Gibbs sampler) [67] and reported that the motif detection ability of PhyME was better than the other algorithms in most cases.

Moses *et al*. [15] developed the algorithm EMnEm that uses EM and a phylogenetic model to find motifs from data involving coregulated genes and orthologous sequences. This algorithm assumes that the input sequences are completely aligned, however, such an assumption may not be suitable for species at relatively large evolutionary distance such as human and mouse.

Siddharthan *et al*. [16] developed the algorithm PhyloGibbs that combines the motif finding strategies of phylogenetic footprinting and Gibbs sampling into one integrated Bayesian framework. PhyloGibbs runs on arbitrary collections of multiple local sequence alignments of orthologous sequences. The algorithm searches over all arrangements in which an arbitrary number of binding sites for an arbitrary number of transcription factors can be assigned to the multiple sequence alignments. These binding site configurations are scored by a Bayesian probabilistic model that treats aligned sequences by a model for the evolution of binding sites and background intergenic DNA. This model takes the phylogenetic relationship between the species in the alignment explicitly into account. The algorithm uses simulated annealing and MCMC sampling to rigorously assign posterior probabilities to all binding sites that it reports. In tests on synthetic data and real data from five *Saccharomyces *species this algorithm performed significantly better than the algorithms MEME, Gibbs sampler, PhyME and EMnEM.

## Performance evaluations of motif finding algorithms

A large number of motif finding algorithms are available, therefore, users may like to have some guidance in choosing the best tools for their motif finding endeavor. However, it has been a challenging task to conduct studies on performance comparisons of motif finding tools. As mentioned by Tompa *et al*. [57], the difficulty in performance assessment of motif finding tools stems from several sources. The tools have been developed based on varied and complex motif models, and therefore, individual tools may do better on one type of data but do worse on other types of data. Also our incomplete understanding of the biology of regulatory mechanism does not always provide adequate evaluation of underlying algorithms over motif models.

Most authors test their algorithm against a few available algorithms using both biological sequence data and synthetic data sets with planted motifs. Pevzner and Sze [36] tested their combinatorial algorithm SP-STAR with the probabilistic algorithms GibbsDNA (version of Gibbs sampler to work with DNA sequences), Consensus and MEME and reported that SP-STAR performed better than the other three algorithms on short motifs. Sinha and Tompa [28] compared the accuracy of three motif finding algorithms: YMF, MEME and AlignACE. The performance score that they used was: the number of positions, over all sequences where occurrences of the known and reported motifs overlap, divided by the total number of position at which the known or reported motif occurs. The comparison was done on synthetic data with planted motifs as well as on real data sets of coregulated genes from *S. cerevisiae*. YMF was found to be more accurate than the other two algorithms on the *S. cerevisiae *data set.

Tompa *et al*. [57] assessed performance of thirteen motif finding algorithms. The purpose of their assessment was twofold: providing some guidance regarding the accuracy of currently available motif finding tools in various settings, and to provide benchmark data sets for assessing future tools. Based on the fact that little is known about most transcription factors and their target binding sites, even in well studied organisms, the authors included those computational tools designed for the discovery of novel regulatory elements, where nothing is assumed a priori of transcription factor or its binding sites. For these tools, a user provides a collection of regulatory regions of genes that are believed to be coregulated, and the tool identifies motifs that are statistically overrepresented in these regulatory regions. The thirteen motif-discovery tools assessed by the authors were AlignACE, ANN-Spec [68], Consensus, GLAM [69], Improbizer [70], MEME, MITRA, MotifSampler, Oligo/Dyad-Analysis, QuickScore [71], SeSiMCMC [72], Weeder and YMF. They created data sets containing known binding sites to test these tools. Without revealing the known binding sites, each author with specific expertise on a particular tool then ran that tool on these data sets. Experts were chosen to test each tool so that none would be put at the disadvantage of being run with an uninformed setting of its parameters. The expert predictions were then compared with the known binding sites, using various statistics to assess the correctness of the predictions.

For binding sites they used the TRANSFAC database [73] to choose real transcription factors, their known binding sites, and the positions and orientations of those binding sites. Each such transcription factor had one data set of sequences. Each such data set consisted of one of three different types of background sequence, with the transcription factor's known binding sites planted at their known positions and orientations. The three types were (1) the binding sites' real promoter sequences, (2) randomly chosen promoter sequences and (3) sequences generated by a Markov chain of order 3. They used several statistics to assess the performance of each tool on each data set at the nucleotide level as well as at the site level using the information on the known binding sites and the set of predicted binding sites by the tool.

Data revealed that the absolute measures of correctness of the programs were low. For example, site sensitivity was at most 0.22 and correlation coefficient was at most 0.20. Site sensitivity is the statistics that gives the fraction of known sites that are predicted while the correlation coefficient is the Pearson product-moment correlation between two sets of positions (known nucleotide positions and predicted nucleotide positions). The authors warned that this assessment should not be taken as an indictment of computational methods for prediction of regulatory element for a very great number of reasons. Most importantly, the underlying biology of regulatory mechanisms is very incompletely understood. We lack an absolute standard against which to measure the correctness of tools.

The results of the comparison experiments showed that the tool Weeder outperformed the other tools in most domains and by most measures in this assessment. The authors believe that some part of Weeder's success is due to judicious choices regarding when to predict no motif in a data set: Weeder was run in a "cautious mode", where only the strongest motifs were reported. A few small exceptions to Weeder's domination were that the SeSiMCMC did somewhat better on the fly data set and the MEME3 (a variation of MEME) and YMF did somewhat better on the mouse data sets. The authors suggest that biologists should use a few complementary tools in combination rather than relying on a single one and to pursue the top few predicted motifs of each rather than the single most significant motif.

Hu *et al*. [58] also conducted a comprehensive benchmark experiment for performance comparisons of five sequence-based motif finding algorithms using large datasets generated from *E. coli *RegulonDB. The authors for this study have pointed out how their work differs from the benchmark experiments of Tompa *et al*. [57] for performance comparisons of motif finding algorithms. In the study by Tompa *et al*. [57], the algorithm developers were allowed to fine-tune the running parameters and reported the best results while Hu *et al*. [58] allowed minimal parameter tuning during performance evaluation. They also suggest that performance evaluation based only on the predictions with the highest score has the risk of penalizing some practically effective algorithms, since in many cases the predicted motifs with the highest score are not the motif with highest accuracy.

Five algorithms assessed by the authors were AlignACE, MEME, BioProspector, MDScan and MotifSampler. The authors defined a set of prediction performance indexes for the algorithms and conducted comparative evaluations of the algorithms in terms of their prediction accuracy, scalability and the reliability of their significance scores with the RegulonDB. The prediction accuracy measures used by these authors were nucleotide level accuracy, binding site level accuracy, and sequence and motif level accuracy. This study showed that the performance of the algorithms tested is quite low, with around 15 to 25% accuracy at the nucleotide level and 25 to 35% at the binding site level for sequences of 400 nucleotides long. However, the algorithms were capable of predicting at least one binding site correctly more than 90% of the time. Among the factors that affect the prediction accuracy, the sequence length was found to be the most critical; the performance of all algorithms degrades significantly as the sequence length increases. In this study, the authors also included an ensemble algorithm for comparison. The ensemble algorithm achieved a better performance than the popular MEME algorithm by 52%.

## Conclusion

Since transcription factors bind to DNA motifs and modulate gene expression, identification of motifs in the promoter region of genes will help understand some aspects of regulation of gene expression. Therefore, biologists and computer scientists have been very interested in identifying computational tools for motif finding. With the advent of availability of large scale genome sequencing and high-throughput gene expression analysis techniques, a large number of motif finding tools have been designed and implemented over the past decade. Our survey of the developments in the area of DNA motif finding algorithms show that diverse approaches such as combinatorial enumeration, probabilistic modeling, mathematical programming, neural networks and genetic algorithms have been employed to develop motif finding tools. Earlier algorithms relied on coexpressed genes and searched for overrepresented motifs. Recent algorithms take advantage of motif's overrepresentation and conservation among orthologous sequences. From this large number of available tools for motif finding, users would like to have guidance in choosing the generally best tool. However, assessment of performance of tools has still been a difficult task. This is mainly because we do not have a clear understanding of the biology of regulatory mechanisms, therefore, we lack an absolute standard against which to measure correctness of tools. Most of the algorithms perform better in lower organisms including yeast as compared to higher organism. Recent algorithms that integrate the motif overrepresentation and cross-species conservation have proven to perform better in higher organism including human [7,14]. We agree with Tompa *et al*. [57] that biologists should use a few complementary tools in combination rather than relying on a single one and pursue the top few predicted motifs of each rather than the single most significant motif, which also reflects the success of Hu *et al*. [58] developed ensemble algorithm.

## Competing interests

The authors declare that they have no competing interests.

## Authors' contributions

Both authors contributed equally in topic selection and in designing the structure and organization of the manuscript. MKD wrote the draft of the manuscript. HKD directed the project and continually reviewed and helped improve the manuscript. Both authors have read and approved the manuscript.

## Supplementary Material

Additional file 1 Some motif discovery algorithms Click here for file
