# Supplementary material for: A survey of DNA motif finding algorithms
Source: BMC Bioinformatics. 2007 Nov 1;8(Suppl 7):S21. doi: 10.1186/1471-2105-8-S7-S21 (PMC2099490; doi:10.1186/1471-2105-8-S7-S21)
Supplement: Additional file 1 — Some motif discovery algorithms [file 1471-2105-8-S7-S21-S1.doc]

Some motif discovery algorithms

| Algorithm | Operating principle | Classification | Reference |
| --- | --- | --- | --- |
| by Galas *et al*. | Enumeration | Word-based | Galas *et al*. [74] |
| by Mengeritsky and Smith | Enumeration | Word-based | Mengeritsky and Smith [75] |
| by Staden | Enumeration | Word-based | Staden [76] |
| EM | Expectation maximization | PSM ^a)^ | Lawrence and Reilly [41] |
| WordUP | Enumeration | Word-based | Pesole *et al*. [77] |
| Gibbs sampler | Gibbs sampling | PSM | Lawrence *et al*. [42] |
| MACAW | Gibbs sampling | PSM | Liu [78] |
| MEME | Expectation maximization | PSM | Bailey and Elkan [29] |
| AlignACE | Gibbs sampling | PSM | Roth *et al*. [30] |
| Oligo-Analysis | Enumeration | Word-based | van Helden *et al*. [23] |
| Consensus | Weight matrix | PSM | Hertz and Stormo [39] |
| Dyad-Analysis | Enumeration | Word-based | van Helden *et al.* [24] |
| WINNOWER | Graph | Other | Pevzner and Sze [36] |
| ANN-Spec | Gibbs sampling | PSM | Workman and Stormo [68] |
| SMILE | Suffix tree | Word-based | Marsan and Sagot [33] |
| Verbumculus | Suffix tree | Word-based | Apostolico *et al*. [79] |
| MobyDick | Dictionary | Word-based | Bussemaker *et al.* [80] |
| YMF | Enumeration | Word-based | Sinha and Tompa [26] |
| Bioprospector | Gibbs sampling | PSM | Liu *et al*. [47] |
| Co-Bind | Gibbs sampling | PSM | GuhaThakurta and Stormo [81] |
| ITB | Enumeration | Word-based | Kielbasa *et al*. [82] |
| Weeder | Enumeration | Word-based | Pavesi *et al.* [34] |
| MotifSampler | Gibbs sampling | PSM | Thijs *et al*. [46] |
| MITRA | Prefix tree/Graph | Word-based | Eskin and Pevzner [35] |
| MDScan | Greedy algorithm | Other | Liu *et al.* [60] |
| Projection | Hashing | Other | Buhler and Tompa [49] |
| Footprinter | Dynamic programming | Other | Blanchette and Tompa [4] |
| MOPAC | Enumeration | Word-based | Ganesh *et al*. [83] |
| DMotif | Enumeration | Word-based | Sinha [84] |
| PhyloCon | Consensus | PF ^b)^ | Wang and Stormo [13] |
| LOGOS | Expectation maximization | PSM | Xing *et al*. [85] |
| EC | Genetic algorithm | Other | Fogel *et al*. [86] |
| GLAM | Gibbs sampling | PSM | Frith *et al*. [69] |
| Improbizer | Expectation maximization | PSM | Ao *et al*. [70] |
| QuickScore | Consensus | PSM | Regnier and Denise [71] |
| SeSiMCMC | Gibbs sampling | PSM | Favorov *et al*. [72] |
| PhyME | Expectation maximization | PSM | Sinha *et al.* [14] |
| OrthoMEME | Expectation maximization | PSM | Prakash *et al*. [12] |
| FMGA | Genetic algorithm | Other | Liu *et al*. [53] |
| PHYLONET | Sequence alignment | PF | Wang and Stormo [7] |
| PhyloGibbs | Gibbs sampling | PSM | Siddharthan *et al*. [16] |
| GIMF | Expectation maximization | PSM | Qi *et al*. [87] |
| WordSpy | Dictionary | Word-based | Wang *et al*. [88] |
| MaMF | Enumeration | Word-based | Hon and Jain [17] |
| EMD | Clustering-based ensemble | Other | Hu *et al*. [59] |
| GibbsST | Gibbs sampling | PSM | Shida [48] |
| MUSA | Biclustering | Other | Mendes *et al*. [89] |
| GAME | Genetic algorithm | Other | Wei and Jensen [90] |
| ALSE | Expectation maximization | PSM | Leung and Chin [91] |
| MotifSeeker | Data fusion and ranking | Other | Peng *et al.* [92] |
| PhyloScan | Scanning | PF | Carmack *et al.* [8] |

a) PSM = Probabilistic sequence model

b) PF = Phylogenetic footprinting
